# Localization Algorithms of Underwater Wireless Sensor Networks: A Survey

**DOI:** 10.3390/s120202026

**Published:** 2012-02-13

**Authors:** Guangjie Han, Jinfang Jiang, Lei Shu, Yongjun Xu, Feng Wang

**Affiliations:** 1 Department of Information and Communication Systems, Hohai University, 200 North Jinling Road, Changzhou 213022, China; E-Mail: jiangjinfang1989@gmail.com; 2 Changzhou Key Laboratory of Sensor Networks and Environmental Sensing, Changzhou 213022, China; 3 Department of Multimedia Engineering, Graduate School of Information Science and Technology, Osaka University, 1-1 Yamadaoka, Suita, Osaka 565-0871, Japan; E-Mail: lei.shu@live.ie; 4 Institute of Computing Technology, Chinese Academy of Sciences, No. 6 Kexueyuan South Road Zhongguancun, Beijing 100190, China; E-Mail: xyj@ict.ac.cn; 5 Naval Academy of Armament, Beijing 100161, China; E-Mail: lionkingwf@hotmail.com

**Keywords:** underwater wireless sensor networks, localization algorithms, centralized, distributed, hybrid

## Abstract

In Underwater Wireless Sensor Networks (UWSNs), localization is one of most important technologies since it plays a critical role in many applications. Motivated by widespread adoption of localization, in this paper, we present a comprehensive survey of localization algorithms. First, we classify localization algorithms into three categories based on sensor nodes’ mobility: stationary localization algorithms, mobile localization algorithms and hybrid localization algorithms. Moreover, we compare the localization algorithms in detail and analyze future research directions of localization algorithms in UWSNs.

## 1. Introduction

The majority of the earth’s surface is covered by water. Various underwater applications have been researched, e.g., environmental monitoring, undersea explorations, disaster prevention, mine reconnaissance, *etc.* [1]. Traditional monitoring systems are expensive and complicated. These equipments utilize individual and disconnected equipments to collect data from their surrounding environments [2,3]. The emergence of UWSNs provides new opportunities to explore the ocean. In UWSNs, conventional large, expensive, individual ocean monitoring equipments are replaced by relatively small and less expensive underwater sensor nodes that are able to communicate with each other via acoustic signals. Many technologies for UWSNs have been researched, e.g., medium access control (MAC) and secure routing protocols, localization technologies and time synchronization schemes. Localization is one of the most important technologies since it plays a critical role in many applications.

Generally, there are three kinds of sensor nodes in UWSNs: anchor nodes, unknown nodes and reference nodes. Unknown nodes are responsible for sensing environment data. Anchor nodes are responsible for localizing unknown nodes. They can acquire their position in advance using GPS systems or artificial arrangement. Reference nodes consist of localized unknown nodes and initial anchor nodes. Localization process of an unknown node can be described as how the node determines its position by limited communication with several anchor nodes or reference nodes using some specific localization technologies.

Although various localization algorithms have been proposed for terrestrial WSNs, they are not suitable for UWSNs [4,5]. The major difference between UWSNs and terrestrial WSNs is the different communication signals. Radio signal propagates at long distances through sea water only at extra low frequencies between 30 Hz and 300 Hz. Low-frequency radio signal requires long antennae and high transmission power. Relatively, acoustic signal attenuates less and travels further. Thus, acoustic signal is more suitable for UWSNs [6]. Acoustic communication channel has its unique characteristics. Hence, the existing localization algorithms for terrestrial WSNs cannot be applied to UWSNs.

Currently, many localization algorithms have been proposed for UWSNs. Researchers in [2,3] classify these localization algorithms into two categories: distributed and centralized localization algorithms, based on where the location of an unknown node is determined. In distributed localization algorithms, each underwater unknown node collects localization information and then runs a location estimation algorithm individually. In centralized localization algorithms, the location of each unknown node is estimated by a base station or a sink node. These two categories are further divided into subcategories of estimation-based and prediction-based algorithms. Estimation-based algorithms use current information to compute the location of a node, while prediction-based algorithms aim at predicting the location of a node at the next time instant, using previous and current location information. Moreover, another early survey paper [7] researched several terrestrial localization algorithms and analyzed their suitability for UWSNs. However, localization algorithms for UWSNs have not been discussed.

In UWSNs, the movement of sensor nodes caused by water currents is inevitable. The classification in [2,3] is not distinct enough without considering the mobility of sensor nodes. Thus, in this paper, we reclassify the localization algorithms based on the mobility of sensor nodes. The rest of paper is organized as follows. In Section 2, we focus on recent localization algorithms from 2006 to 2011 and reclassify the algorithms with a new perspective. In Sections 3, 4 and 5, we present a detailed analysis and comparison of the localization algorithms based on several evaluation criteria, e.g., localization accuracy, energy consumption, *etc.* Finally, the future research direction of UWSNs’ localization algorithms is discussed.

## 2. Classification of Localization Algorithms

### 2.1. Classification Based on Sensor Nodes’ Mobility

**Figure 1.**
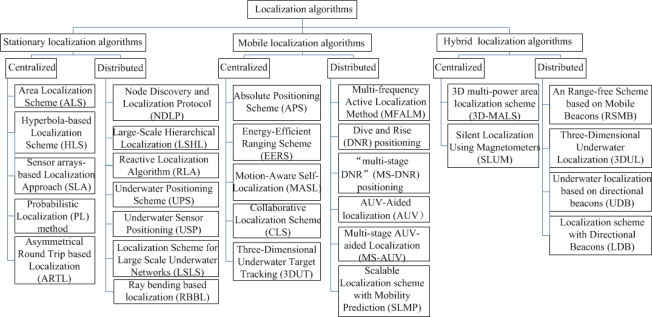
Localization Algorithms Classification in UWSNs.

As shown in Figure 1, localization algorithms are classified into three categories. (1) In stationary localization algorithms, all sensor nodes are static. They are attached to surface buoys or ocean floor units which have fixed locations. (2) In mobile localization algorithms, all sensor nodes are mobile. They freely drift with water currents or use propelled equipments, e.g., Autonomous Underwater Vehicle (AUV), to control their movements. (3) In hybrid localization algorithms, stationary and mobile sensor nodes coexist. These three categories are further divided into subcategories of centralized and distributed localization schemes.

### 2.2. Evaluation Criteria

In this paper, we analyze and summarize typical localization algorithms of each category. Furthermore, the localization algorithms are compared from following aspects:

- Anchor type: Most localization algorithms of UWSNs use anchor nodes to localize unknown nodes. Unfortunately, GPS cannot work well in sub-sea environment. Thus, some researchers propose self-localization algorithms, e.g., the Node Discovery and Localization Protocol (NDLP) in [8,9].
- Ranging method: In UWSNs, in order to take advantage of the slow propagation speed of sound under water, range-based schemes that use ToA (Time of Arrival) and TDoA (Time Difference of Arrival) are suggested for UWSNs [10].
- Message communication: There are two kinds of message communication: silent and active. “Silent communication” is that unknown nodes passively listen messages from neighbor nodes and do not transmit any packets, while “active communication” needs unknown nodes to participate in localization message exchange. Relatively speaking, silent communication is much more energy efficient than active communication.
- Time synchronization: Time synchronization is an important technology for UWSNs. Precise time synchronization is difficult to achieve in underwater environments. Therefore, in most localization algorithms, sensor nodes are assumed to be perfectly synchronized with each other.
- Localization coverage: Maximizing sensing coverage is a fundamental requirement for UWSNs. Many researchers have been focused on the coverage issue, e.g., [11], where the goal is to find a node placement strategy with 100% sensing coverage of a 3D space, while minimizing the number of nodes required for surveillance.
- Localization time: Localization time of any localization algorithm cannot be too long. If localization process needs long time, the calculated locations are different from the real ones, since sensor nodes may move to new places.
- Localization accuracy: Localization accuracy is the most important evaluation criterion. Although some coarse-grained localization algorithms have been proposed, most applications, e.g., target tracking, require high localization accuracy.
- Computational complexity: Due to limited energy resource, computation of localization algorithms should be as simple as possible.
- Energy consumption: Any localization algorithm cannot be feasible if energy consumption is too high.

## 3. Stationary Localization Algorithms

For simplicity, many localization algorithms assume that sensor nodes in UWSNs are stationary. We analyze and compare these stationary localization algorithms is this section.

### 3.1. Centralized Localization Algorithms

In this section, five centralized localization schemes are summarized: (1) Area Localization Scheme (ALS), (2) Hyperbola-based Localization Scheme (HLS), (3) Sensor arrays based localization approach, (4) an Probabilistic Localization Method (PLM) and (5) Asymmetrical Round Trip based Localization (ARTL) algorithm.

#### 3.1.1. Area Localization Scheme (ALS)

For large scale UWSNs, identifying the exact location of every unknown node may not be feasible. Therefore, in [12], an efficient Area Localization Scheme (ALS) is proposed for UWSNs. ALS was firstly proposed for terrestrial WSNs in [13]. This scheme estimates the position of every unknown node within a certain area rather than its exact location. The main responsibility of anchor nodes is to send out signals with different levels of power to localize unknown nodes. Unknown nodes simply listen to the signals and record the anchor nodes’ IDs and their corresponding power levels. Together with collected data, the recorded information is sent to sink node. Sink node is assumed to know the positions of all anchor nodes and their respective transmitted power levels. Therefore, with proper signal propagation algorithms, sink node is able to draw out the map of areas divided by all the anchor nodes’ transmitting signals. Then, sink node can localize unknown nodes.

The advantages of ALS are its being range-free and having no synchronization requirement. Moreover, all complex calculations are handled by the powerful sink node instead of unknown nodes. This reduces energy consumption of unknown nodes and extends the lifetime of network. Localization coverage can be appropriately broadened by adjusting the transmitting power of anchor nodes. However, as a coarse localization scheme, ALS is not convenient for applications that require accurate and instant location information. In addition, ALS incurs high communication overhead and energy consumption. ALS handles localization by assuming that sink node knows the positions of all anchor nodes and their respective transmitted power levels. This assumption reduces flexibility of the network. After deploying the network, it is not possible to change positions of anchor nodes. If the positions of anchor nodes are changed by water currents, the performance of ALS decreased greatly.

#### 3.1.2. Hyperbola-Based Localization Scheme (HLS)

**Figure 2.**
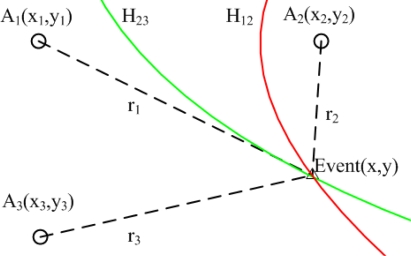
The Hyperbola-Based Localization Scheme.

In [14], the authors proposed a Hyperbola-based Localization Scheme (HLS). Instead of using the commonly adopted circle-based detection and least squares algorithm based location estimation, the proposed scheme utilizes the hyperbola-based approach for localization and a normal distribution for estimation error modelling and calibration. As shown in Figure 2, when any unknown node detect one event, it will report the event to anchor nodes immediately. Anchor nodes *A*_1_ and *A*_2_ receive the event at time *t*_1_ and *t*_2_. The difference between *t*_1_ and *t*_2_ is a constant, in other words, the difference between the corresponding distance *r*_1_ and *r*_2_ is a constant. Based on the property of hyperbola, the unknown node is localized on a hyperbola *N*_12_. Similarly, another hyperbola *N*_23_ is plotted in the same figure. More curves can be added when more anchor nodes are involved in localization. The intersection of any two curves gives an estimation position of unknown node. In practical applications, the obtained set of intersection points may not coincide or converge well. In this case, algorithms like least squares can be adopted to further improve localization accuracy.

Compared with the circle-based approach, HLS is more robust against distance measurement error and can localize more unknown nodes. Since two hyperbolas always intersect with each other with one cross point, while two circles will likely intersect with either two or zero cross point(s); there is very little chance to have only one cross point. Simulation results show that localization accuracy of HLS is better than that of the least squares algorithm. In general, HLS is more suitable for accurate localization in UWSNs. However, in HLS, sensor nodes are required to send long-range signals (around 1,000 meters). Thus, excessive energy is consumed. Furthermore, anchor nodes need to be stationary and hence HLS is not extendable to mobile UWSNs.

#### 3.1.3. Sensor Arrays-Based Localization Approach (SLA)

In [15], Maximum-Likelihood Source Localization approach (MLSL) is proposed. The UWSN in [15] consists of sensor arrays. Each sensor array is equipped with an array of sensor nodes that are attached to the sensor array via wired connections. Each target waiting to be localized periodically emits a narrow-band acoustic signal. For each sensor array, using the negative log-likelihood function, sensor nodes which have received the signal can obtain the target locations and signal amplitudes. The maximum likelihood estimate of the target location is obtained based on the global likelihood function, which is the sum of the local likelihood function. MLSL approach does not need distance measurement and time synchronization. Computation overhead of sensor nodes and targets are low, while communication overhead and energy consumption are high as all the local likelihood functions are forwarded to a fusion center. Moreover, the local wired and global wireless network architecture is not feasible for large scale UWSNs.

Another localization method using sensor arrays has been proposed in [16] named Energy-based Source Localization Method (ESLM). ESLM uses the acoustic signal energy attenuation model to estimate distance. The method reduces energy consumption of accurate time synchronization among nodes. However, when the distance between two nodes is too far (greater than 8 meters), localization result based on the acoustic signal energy attenuation model is more sensitive to noise and it is not reliable.

#### 3.1.4. An Probabilistic Localization Method (PLM)

To mitigate distance measurement error in localization process, multi-iteration measurement and least squares scheme are often adopted in terrestrial applications. However, in underwater applications, the multi-iteration scheme is not practical due to high communication cost. Meanwhile, it has been observed that the probability distribution of distance measurement error often follows a certain pattern, which can be utilized to further improve localization accuracy. In [17], both uniform error distribution and normal error distribution are considered. Then, an Probabilistic Localization Method (PLM) is proposed to improve localization accuracy.

PLM method consists of two steps. In the first step, every two neighboring anchor nodes calculate positions of unknown nodes using a circle-based or hyperbola-based approach. The second step is to determine the final locations of unknown nodes by using the probability distribution of distance measurement error. Localization coverage and accuracy can be improved by utilizing more anchor nodes. However, computation complexity and energy consumption increase greatly. Simulation results indicate that PLM method can significantly improve localization accuracy. Compared with other methods, e.g., minimum mean absolute error (MMAE) and minimum mean squared error (MMSE) based statistical approaches, PLM method requires less information exchange. However, the two ideal error distributions may not make sense in real UWSNs environment.

#### 3.1.5. Asymmetrical Round Trip Based Localization (ARTL) Algorithm

**Figure 3.**
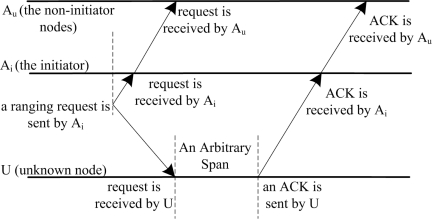
The Asymmetric Round-Trip Scheme.

An Asymmetrical Round Trip based Localization (ARTL) algorithm is proposed in [18]. ARTL assumes that anchor nodes can receive their own packets while unknown nodes cannot do. First, the basic ranging scheme is implemented to get distances between anchor nodes and unknown nodes. As shown in Figure 3, unlike existing symmetrical round-trip schemes [19,20], the ranging scheme here is asymmetric; that is, to estimate the distance between an anchor node and unknown node *U*, the initiator (the anchor node that starts the ranging task) *A_i_* first broadcasts a ranging packet, which is received by both itself and *U*, as well as other non-initiator nodes *A_u_*. Then after an arbitrary period, *U* responds an ACK to *A_i_*. This ACK will not be received by *U* itself, but only received by *A_i_* as well as *A_u_*. Based on the time difference of arrival signals, the distance can be calculated. Together with collect data information, the calculated distances are sent to the base station which launches localization task. Thus, no time synchronization is required in the entire process and no complex computations are handled by anchor nodes and unknown nodes. Moreover, ARTL does not require unknown nodes to immediately reply after receiving the ranging broadcast, so *U* can respond ACK whenever it is convenient.

Compared with other existing symmetrical round-trip schemes, ARTL needs very little communication overhead. For example, using *s* anchor nodes to localize *l* unknown nodes, traditional symmetrical round-trip schemes need *s × l* ranging requests from anchor nodes and another *s × l* replies from unknown nodes. Therefore, 2*s × l* messages are exchanged to finish all the related ranging tasks, which consumes too much energy. However, in ARTL, only *l* + 1 broadcast packets are needed to obtain all the required ranging related measurements. One is from the initiator and the other *l* are from unknown nodes. Furthermore, ARTL is easy to extend. Localization coverage can be enlarged by adding new anchor nodes. Whenever a new anchor node is added to the network, neither program modifications to other existed nodes nor additional calculations in the ranging process are needed. In general, localization accuracy become better and better with increasing number of anchor nodes. However, more anchor nodes incurs more packet exchanges and higher communication overhead. The main drawback of ARTL is long localization time. Moreover, much energy is consumed to send the calculated distances to the base station.

### 3.2. Distributed Localization Algorithms

In this section, seven distributed localization schemes are summarized: (1) Node Discovery and Localization Protocol (NDLP), (2) Large-Scale Hierarchical Localization (LSHL) approach, (3) Reactive Localization Algorithm (RLA), (4) Underwater Positioning Scheme (UPS), (5) Underwater Sensor Positioning (USP), (6) Localization Scheme for Large Scale underwater networks (LSLS) and (7) Ray Bending based Localization (RBL).

#### 3.2.1. Node Discovery and Localization Protocol (NDLP)

**Figure 4.**
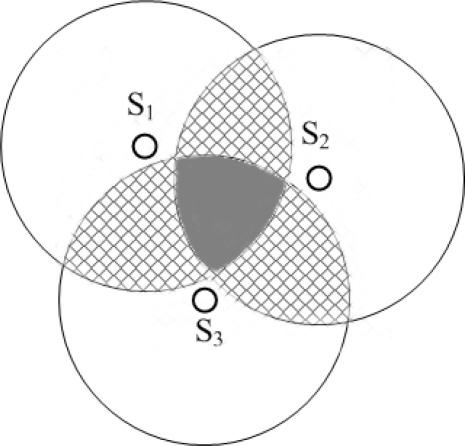
The Node Discovery and Localization Protocol.

In [8,9], the authors proposed a Node Discovery and Localization Protocol (NDLP) to manage sub-sea localization. As shown in Figure 4, NDLP starts with one seed node (primary seed *S*_1_) with known position. The primary seed node is capable of determining the relative positions of neighboring nodes. A second seed node, *S*_2_ is then chosen by *S*_1_. *S*_2_ is the most distant node within the communication range of *S*_1_. The advantage of choosing the farthest node as the second seed node is that a larger area can be covered more quickly. A third seed node *S*_3_ is chosen from those nodes that lie in both communication ranges of *S*_1_ and *S*_2_, and has the maximum summation distance from *S*_1_ and *S*_2_. Each node in the overlapping region (the grey area in Figure 4) is able to calculate the relative location using a simple triangulation technique. The nodes in the cross-hatched region in Figure 4 can only obtain two distance measures from seed nodes. In order to localize the nodes in the cross-hatched region, a fourth seed node is selected based on four algorithms, Farthest/Farthest Algorithm, Farthest/Nearest Algorithm, Nearest/Farthest Algorithm and Nearest/Nearest Algorithm.

NDLP is an anchor-free and GPS-less algorithm. Large scale of unknown nodes can be localized by continuously selecting seed nodes. However, NDLP has some serious problems. First, the node discovery phase needs much communication overhead. Each node participates in massage exchange to select seed nodes. Hence, energy consumption of node discovery is high. Second, the seed node’s selection process takes long time, hence localization time is long. Moreover, unknown nodes’ relative coordinates calculated based on the seed nodes’ positions are not accurate. In some areas, if nodes are much sparsely deployed or become sparser due to some movements, then it is possible that very few or even no node can be selected as seed node. It is shown that NDLP is not suitable for sparse and mobile UWSNs. Furthermore, in mobile UWSNs, repeating the node discovery each time the topology changes is unaffordable.

#### 3.2.2. Large-Scale Hierarchical Localization (LSHL) Approach

In [21,22], the authors proposed a Large-Scale Hierarchical Localization (LSHL) approach. Surface buoys drift on water surface and get their locations from GPS. Anchor nodes can directly communicate with the surface buoys to get their absolute positions. Unknown nodes cannot directly communicate with the surface buoys but can communicate with anchor nodes to localize themselves. The whole localization process is divided into two sub-processes: anchor node localization and unknown node localization.

Anchor node localization is not discussed in [21,22]. During the unknown node localization process, there are two types of nodes: reference nodes and non-localized nodes. Each node (including reference nodes and non-localized nodes) periodically broadcasts a beacon message, containing its ID. All the neighboring nodes which receive this beacon message can estimate their distances to this node using measurement techniques, such as ToA. Besides, each non-localized node keeps a counter, *m*, of the reference nodes to which it knows the distances. Once the localization process starts, each non-localized node keeps checking *m*. If *m <* 4, the non-localized node broadcasts a localization message which contains all its received reference nodes’ locations and its estimated distances. Besides, the non-localized node uses the 3-dimensional Euclidean distance estimation approach to estimate its distances to more non-neighboring reference nodes. After this step, the set of its known reference nodes is updated. Correspondingly, *m* is updated and the node returns to the *m*-checking procedure. Until *m ≥* 4, the non-localized node selects 4 reference nodes with the highest confidence values to localize itself. After the non-localized node is localized, it computes confidence value *η*. If *η* is larger than or equal to the confidence threshold λ, the non-localized node labels itself as a new reference node. Simulation results show that localization coverage and average communication overhead are not affected much by the mobility of sensor nodes, while localization error increases noticeably with the moving speed of sensor nodes. Thus, LSHL cannot provide accurate localization accuracy in mobile UWSNs. Moreover, LSHL has inherent problem of confidence threshold λ, which can affect its performance.

#### 3.2.3. Reactive Localization Algorithm (RLA)

In [23], Reactive Localization Algorithm (RLA) is proposed. Instead of localizing every single node in the network, RLA localizes a node that detects an event. Once a sensor node detects an event, RLA which consists of two steps starts. The first step is to find anchor nodes. The sensor node first broadcasts a hello message with its ID and energy level to its neighbors. By the K-Node Coverage Algorithm, at least 4 non-coplanar anchor nodes are found. The second step is reactive localization of the sensor node. Once the selected anchor nodes receive localization request message, they reply with their location information. The sensor node hence localizes itself by quadrilateration. Due to additional process for anchor nodes’ localization, energy consumption and communication overhead of RLA are high. Furthermore, accumulated localization error exists.

#### 3.2.4. Underwater Positioning Scheme (UPS)

**Figure 5.**
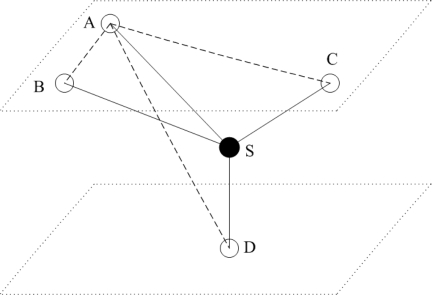
The Underwater Positioning Scheme.

Based on the Time-based Positioning Scheme (TPS) [24] for outdoor WSNs, the authors in [25,26] proposed Underwater Positioning Scheme (UPS). UPS consists of two steps. The first step is to detect the time differences of arrival signals. Then, the time differences are transformed into range differences. As illustrated in Figure 5, *A* is the master anchor node, which initiates a beacon signal every *T* seconds. *B*’s transmission starts after it receives *A*’s beacon signal. *C*’s transmissions starts after it receives beacon signals of both *A* and *B*. In addition, *D*’s transmission starts after it receives beacon signals of *A*, *B* and *C*. After *S* get all the beacon signals, the time difference of arrival signals is calculated and converted to range difference. In the second step, trilateration is performed to localize unknown nodes.

UPS requires no time synchronization and has low computation overhead. As evidenced by simulation results, UPS has low localization error. It is applicable to both localization and navigation in UWSNs. Moreover, few anchor nodes are needed to perform 3D localization. However, sensor nodes outside the four anchor nodes’ communication range cannot be localized. In addition, UPS does not take into account the impact of transmission failures, which is highly likely because underwater acoustic channels are unreliable. If anchor node *B* cannot receive the beacon signal of the master anchor node *A*, it will not send its own beacon signal. Then, *C* and *D* will keep waiting until receive beacon signal from node *B*. Therefore, in [27], an Enhanced-UPS (E-UPS) is proposed. On one hand, a time-out value is designed for the maximum waiting time of the anchor node messages. On the other hand, to overcome the limitation of relying on four anchor nodes, E-UPS is extended to use all anchor nodes. It is observed that up to 16% of the network containing anchor nodes is not localizable [28]. Therefore, a Wide Coverage Positioning System (WPS) [28] is proposed to address this limitation using five anchor nodes.

#### 3.2.5. Underwater Sensor Positioning (USP)

**Figure 6.**
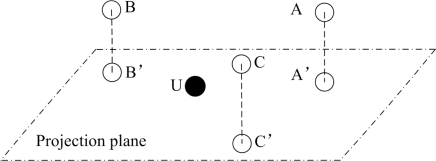
The Underwater Sensor Positioning.

Underwater Sensor Positioning (USP) scheme is proposed in [29–31] for sparse 3D UWSNs, as shown in Figure 6. At least three anchor nodes are included in the network. To simplify the process of endowing anchor nodes with their positions, they are placed at the surface as GPS-enabled buoys. In USP scheme, unknown nodes get their depth information by pressure sensors. The depth information is used to transform the 3D underwater positioning problem into its 2D counterpart via the projection technique. For example, consider an unknown node *U* that needs to compute its position within a 3D oceanic deployment area. It is assumed that node *U* is within communication range of three anchor nodes *A*, *B* and *C* located at known positions (*x_A_*, *y_A_*, *z_A_*), (*x_B_*, *y_B_*, *z_B_*), (*x_C_*, *y_C_*, *z_C_*), respectively. Given *U*’s depth information *z_U_*, node *A* is projected as node *A′* located at position (*x_A_*, *y_A_*, *z_U_*). Nodes *B* and *C* are projected as nodes *B′* and *C′* located at position (*x_B_*, *y_B_*, *z_U_*) and (*x_C_*, *y_C_*, *z_U_*). After three anchor nodes *A′*, *B′* and *C′* have been projected, elegant localization methods such as simple bilateration can be employed to localize node *U*.

USP scheme first uses depth information to localize unknown nodes in sparse 3D UWSNs and has low storage and computation requirements. USP scheme seems simple by introducing pressure sensors. However, installing pressure sensors increases energy consumption per unknown node, hence decreasing the life time of the UWSNs. Moreover, USP scheme has low localization success. In addition, each unknown node consumes much energy to map the available anchor nodes on the horizontal plane it resides on. Furthermore, unknown nodes in deep water cannot be localized because they are without the anchor node’s communication range.

#### 3.2.6. Localization Scheme for Large Scale underwater networks (LSLS)

Integrating UPS and USP, a Localization Scheme for Large Scale underwater networks (LSLS) is proposed in [32], where any localized node can work as reference node to further localize neighbor nodes. Similar to RLA and LSHL, LSLS is a hierarchical localization approach. LSLS includes three phases: sea surface anchor localization, iterative localization, and the complementary phase. In the first phase, three surface anchor nodes send their beacon messages sequentially to calculate the range difference as described in UPS. Then, unknown nodes are localized as described in USP. In the second phase, certain localized nodes are selected to serve as reference nodes. More unknown nodes are localized as described in the first phase. If a node fails to be localized in the first two phases, it can initiate a location request in the third phase. A new group of reference nodes are then selected to localize the unlocalized node.

Similar operations in the first two phases are applied in [33]. However, the remaining unlocalized nodes are not considered in [33]. Compared with UPS, LSLS can localize a large-scale UWSN with short-range acoustic communication. Moreover, LSLS localize more unknown nodes than USP does. However, more energy and communication overhead are consumed to implement the three phases localization.

#### 3.2.7. Ray Bending Based Localization (RBL)

In all the above mentioned localization algorithms, the path of sound rays is assumed to be straight lines. However, because of the depth dependent sound speed, the path of sound rays bends in water. This causes deterioration in performance of the above algorithms when they are deployed in real UWSNs. In [34], authors present a Ray Bending based Localization (RBL) method that accounts for ray bending in water. With the assumption of straight line trajectory and constant velocity, constant range interval surface is spherical. For the ray tracing model [35], constant range interval surface is not spherical; but it retains axial symmetry about the longitudinal axis containing anchor node, since velocity varies only with depth. Simulation study shows that localization using the ray bending model out-performs that using straight line models.

### 3.3. Summary

As shown in Table 1, in this section, we compare the stationary localization algorithms from following aspects:

- Anchor type: Most stationary localization algorithms use anchor nodes to localize unknown nodes except NDLP.
- Ranging method: ALS and MLSL are range-free localization algorithms. HLS, UPS and LSLS use TDoA to obtain distances between anchor and unknown nodes. LSHL and RBBL measure distance by one-way ToA and two-way ToA, respectively. ARTL provides the asymmetrical round-trip strategy to calculate distance.
- Message communication: Unknown nodes participate in the message communication in most algorithms except in USP and USP, where they stay “silent”.
- Time synchronization: Generally speaking, accurate time synchronization introduces more energy consumption and hardware cost. Therefore, time synchronization-free are more suitable. Only HLS and LSHL require time synchronization.
- Localization coverage: Localization coverage of RLA, UPS, USP and RBBL is small. Only the unknown nodes within the communication range of anchor nodes can be localized. However, localization coverage can be appropriately broadened by adjusting the transmitting power (ALS) and using hyperbola-based scheme instead of circle-based one (HLS and PL). Also, some algorithms have been proposed for large scale UWSNs by adding more anchor or seed nodes (ML, ARTL and NDLP) or using hierarchical localization approach (LSHL and LSLS).
- Localization time: In general, large scale localization (LSHL and LSLS) need longer time. ARTL and NDLP requires long time to complete the asymmetrical round-trip strategy and select seed nodes, separately.
- Localization accuracy: LSHL, RLA and LSLS are hierarchical localization introducing inevitable accumulated error. ALS provides a coarse location estimation of an unknown node within a certain area. NDLP is an anchor-free scheme. Therefore, their localization accuracy is low.
- Computational complexity: Most centralized algorithms are computationally light. In contrast with centralized algorithms, distributed algorithms’ computational complexity are often higher.
- Energy consumption: Unknown nodes’ energy consumptions in most stationary localization algorithms are relatively high. Unknown nodes in centralized localization algorithms consume high energy to send information to a central node or sink node, and in distributed localization algorithms they consume high energy to localize themselves.

## 4. Mobile Localization Algorithms

In real application environment of UWSNs, such as environment monitoring, many unknown nodes are mobile. They float freely with water currents. In this section, mobile localization algorithms are introduced.

### 4.1. Centralized Localization Algorithms

In this section, five centralized localization schemes are surveyed: 1) Absolute Positioning Scheme (APS), 2) Energy-Efficient Ranging Scheme (EERS), 3) Motion-Aware Self-Localization (MASL) scheme, 4) Collaborative Localization Scheme (CLS) and 5) Three-Dimensional Underwater Target Tracking (3DUT) Scheme.

#### 4.1.1. Absolute Positioning Scheme (APS)

**Figure 7.**
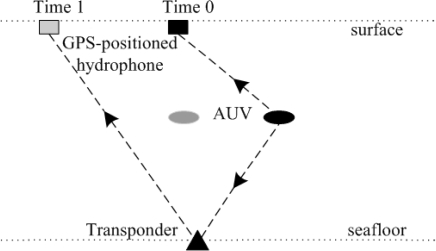
The Absolute Positioning Scheme.

Autonomous Underwater Vehicles (AUVs) provide researchers with new forms of access to ocean. Exploiting AUVs requires AUVs’ accurate position information. Thus, in [36], an Absolute Positioning Scheme (APS) is proposed to localize an AUV. As shown in Figure 7, an AUV transmits an interrogation pulse at a fixed rate. The signals received at the surface consist of a direct signal from the AUV and a reply from each of the transponders. The time difference of arrivals along with depth measurements from an onboard pressure sensor are used to localize the AUV.

APS is proposed to localize one AUV and localization coverage is limited by the acoustic interrogation pulse. Both the AUV and the ship are moving, so the time difference of signal arrivals is not accurate enough. Furthermore, using the GPS-positioned hydrophone aboard the ship to localize AUV incurs high hardware cost and high energy consumption. Therefore, another AUV localization system is proposed in [37], namely Particle Filter [38] based Localization System (PFLS). PFLS is implemented onboard the AUV to localize itself in real-time using ranging information obtained from an UWSN. The AUV is equipped with an acoustic modem allowing it to communicate with the surrounding sensor nodes. Based on the round trip time, the AUV can determine its distances to neighbor nodes. Compared with APS, PFLS are much more efficient.

#### 4.1.2. Energy-Efficient Ranging Scheme (EERS)

In stationary UWSNs, unknown nodes communicate with anchor nodes to estimate the distances between them, from which their positions can be deduced. However, in mobile UWSNs, because of sensor nodes’ uncontrollable mobility, it is impractical to assume that unknown nodes are always in the communication range of fixed anchor nodes. In order to localize a swarm of sensor-equipped drifters that float freely with ocean currents, an Energy-Efficient Ranging Scheme (EERS) is proposed in [39].

First, range can be deduced from the one-way time of exchange message arrival measurement. This step is called Sufficient Distance Map Estimation (SDME). SDME consists of a synchronization step (SDME-S), followed by a distance estimation step (SDME-D). SDME-S is a synchronization-data collection algorithm to achieve time synchronization. SDME-D is a two step distance estimation process. During distance estimation, not all the nodes need to broadcast localization message. Therefore, SDME-D first selects the subset nodes that need to broadcast localization message. The localization based on EERS is an anchor-free self-localization scheme, which can be extended for large scale UWSNs. Although time synchronization is required to handle the one-way ToA, SDME-S can tackle the time synchronization problem efficiently. SDME does not need to be “a full localization” scheme. While submerged, the drifters only need to collect distance estimates. The positions can be calculated after the mission is over. Therefore, energy consumption for position calculation can be saved and complex computation is avoided. However, the subset nodes selection requires a single broadcast per node, which consumes huge energy and prolongs localization time.

#### 4.1.3. Motion-Aware Self-Localization (MASL) Scheme

In wireless communication process, in order to avoid excessive collisions occurring over a shared channel, the gathering of ranging information actually occurs over a short time epoch *T*. The problem is that mobility causes the node positions to change significantly during epoch *T*. Therefore, in [40], the authors proposed a Motion-Aware Self-Localization (MASL) scheme for mobile UWSNs. During MASL, nodes first perform ranging with their neighbors to get all the distance estimates in the localization epoch. Then, all distance estimates are sent to a central station and processed offline. An iteration algorithm is started to obtain the positions. At each iteration, the algorithm refines position distributions by dividing the area of operation into smaller grids, selecting the area in which the node resides with high probability and using it in the next iteration.

MASL is a centralized anchor-free localization algorithm, which reduces unknown nodes’ computational burden and can be used to localize large scale UWSNs. The problem of MASL is that the iterative algorithm cannot provides real-time location information. Real applications always need UWSNs to do online monitoring and provide real-time location information. Simulation results show that, compared with a robust self-localization algorithm, named Multi-dimensional Scaling (MDS) [41], MASL localizes 70% of nodes with error lower than that of MDS.

#### 4.1.4. Collaborative Localization Scheme (CLS)

**Figure 8.**
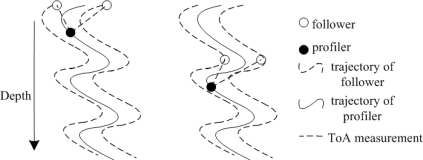
The Collaborative Localization Scheme.

Collaborative Localization Scheme (CLS) for mobile UWSNs is proposed in [42], where nodes collaborate to determine their positions autonomously without using long range transponders on surface buoys or ships. Starting at the surface where the network is deployed, sensor nodes use buoyancy control to descend deeper into ocean. Once a maximum desired depth is reached, they travel back to the surface. While nodes are descending, although they know their depth by pressure sensors, their positions in the other two dimensions change continuously due to the motion induced by currents. In order to track the descending nodes, they are classified into two categories: profilers and followers. Initially, all nodes are at the surface, so that their positions can be obtained by GPS. A profiler travels to a depth first. Then, followers travel the trajectory of the profiler. All the nodes descend with the same speed. The profiler’s location is a prediction of the followers’ future locations. The followers get the profiler’s location by ToA technique, as shown in Figure 8.

CLS is an anchor-free and cost effective self-localization strategy that does not require prior node planning. The drawback of CLS is its architectural dependence; for a sparse or non-homogenous network, the performance of CLS can be affected significantly. Moreover, time synchronization is required. In order to get higher localization accuracy, the profilers have to stay closer to the followers. Otherwise, the profilers going out of communication range of the followers results in localization failures.

#### 4.1.5. Three-Dimensional Underwater Target Tracking (3DUT) Scheme

**Figure 9.**
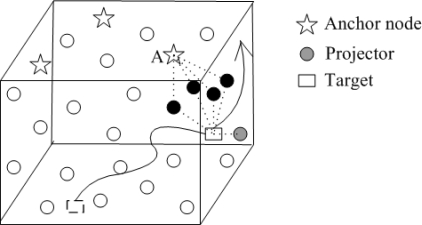
The Three-Dimensional Underwater Target Tracking.

A Three-Dimensional Underwater Target Tracking (3DUT) scheme is proposed in [43]. As shown in Figure 9, at least three anchor nodes float at the surface of water. One of these nodes is the sink (node *A*) which collects the information from underwater sensor nodes and carries out the calculations. The black nodes collect and send information from the target to the sink. The gray node is the designated projector node. 3DUT is a two phase algorithm. During the first phase, Passive Listening, sensor nodes listen to the underwater environment for potential targets. The second phase of the algorithm, Active Ranging, is to localize the target. 3DUT selects a projector node which sends pings periodically. The target is assumed to be a point target so that the echoes are radiated isotropically. Once the echo is received by the projector, it calculates its distance to the target and transmits to the sink node. Sink node uses trilateration to localize the target. The location and the calculated velocity of the target are then exploited to achieve tracking. Depending on the results of the calculations, sink node selects a new projector node.

To save energy, the nodes which are not located at the network edge have low duty cycles. The nodes which are at the boundary of the sensing region have higher duty cycles in order to detect the target entering into the sensing region immediately. Therefore, to avoid rapid energy depletion of boundary nodes due to continuous surveillance, 3DUT employs an adaptive procedure to find, designate, and activate new boundary nodes. Furthermore, 3DUT does not depend on the number of nodes. The algorithm runs even if the number of sensor nodes changes. However, 3DUT can only track one target at a time. Moreover, the tracking accuracy is heavily influenced by the target’s velocity.

### 4.2. Distributed Localization Algorithms

In this section, six distributed localization schemes are surveyed: (1) Multi-frequency Active Localization Method base on TDoA (MFALM), (2) Dive and Rise (DNR) positioning scheme, (3) “multi-stage DNR” (MS-DNR) positioning scheme, (4) AUV-Aided localization technique, (5) Multi-stage AUV-aided Localization (MS-AUV) scheme and (6) Scalable Localization scheme with Mobility Prediction (SLMP).

#### 4.2.1. Multi-Frequency Active Localization Method Base on TDoA (MFALM)

In mobile UWSNs, sensor nodes’ locations are changed at any time. Location information at a time cannot serve as a reference at the next time. Moreover, in general, only the location information of sensor nodes which detect events is useful for UWSNs. Therefore, it is not necessary to localize all sensor nodes in network. We only need to localize sensor nodes which detect events. In [44], the authors propose a Multi-frequency Active Localization Method base on TDoA (MFALM). There are three types of nodes: buoy nodes, relay nodes and ordinary nodes. After the network is deployed, buoy nodes firstly localize themselves using GPS, and periodically broadcast localization information with low-frequency acoustic signals. Relay nodes communicate with each other with low-frequency acoustic signals to divide the network into multiple localization domains and calculate the value of max hops for each domain. Ordinary nodes which detect event open low-frequency signal receiving devices to receive localization information from buoy nodes and localize themselves. At the same time, the ordinary nodes open high-frequency signal sending devices to broadcast MRP (Message Report Package). MRP contains the detected events and the locations of the ordinary nodes. All ordinary nodes which receive the MRP need to relay the packages and decrease the value of max hops by 1. The package broadcasting stops when the value of max hops is 0 or the MRP is received by any relay node. Relay nodes send the received MRP to buoy nodes for further disposal. After disposing the MRP, buoy nodes will respond ACK to ordinary nodes. Then, the ordinary nodes go to sleep until detecting new event.

#### 4.2.2. Dive and Rise (DNR) Positioning Scheme

In [45], an interesting idea of Dive and Rise (DNR) positioning is presented. DNR anchor nodes are used to replace static ones. Each DNR anchor node is equipped with GPS. While sinking and rising, they broadcast their positions. Unknown nodes are localized by passively listening to DNR anchor nodes’ messages. Range measurement is done by using one-way ToA. After hearing from several anchor nodes, unknown nodes estimate their coordinates.

DNR scheme reduces communication overhead and energy consumption by the passively listening method. Unknown nodes spend energy only in receiving and processing localization message. Furthermore, DNR scheme can localize unknown nodes in deep water. However, DNR anchor nodes diving and rising takes longer time than message propagation. Therefore, localization performance heavily depend on frequency of location updates and number of anchor nodes. Compared with LSHL, DNR has higher localization accuracy and less energy consumption and communication overhead [46].

#### 4.2.3. “Multi-Stage DNR” (MS-DNR) Positioning Scheme

To improve performance of DNR, the authors proposed “multi-stage DNR” (MS-DNR) [47] to speed up the localization process at cost of less accuracy and more messaging. Once unknown nodes become localized, they start to act as reference nodes. Therefore, unknown nodes lying out of the communication range of DNR anchor nodes can be localized by the localized unknown nodes. Similar iterative schemes such as LSHL have been proposed. Communication overhead and energy consumption of MS-DNR are relatively high due to the iterative scheme. For this reason, MS-DNR is less energy-efficient than DNR. Compared with DNR, MS-DNR uses a more realistic underwater mobility model, named Meandering Current Mobility (MCM) model, which has been studied in [48]. The model considers sensor nodes’ movement by the effect of meandering sub-surface currents and vortexes. Unlike previous works where nodes are deployed in a small bounded geographic domain, the domain model in MS-DNR is representative of a large coastal environment spanning several kilometers. In this case, assuming sensor nodes uniformly distributed over the large domain is unrealistic. Therefore, the authors consider an initial deployment of sensor nodes in a small subarea where they are released and thereafter move according to the mobility model. A kinematic approach [49–51] is employed to represent the mobility of underwater sensor nodes drifting with subsurface currents. To the best of our knowledge, this is the first physically-inspired mobility model used in the analysis of mobile UWSNs.

The major drawback of the DNR and MS-DNR scheme is the high expense of DNR anchor nodes. There are 25 DNR anchor nodes in 1 km × 1 km × 1 km underwater area, so 25 GPS and 25 moving equipments are needed, which is very expensive. Moreover, under actual underwater environments, the DNR anchor nodes are strongly affected by the surface currents, which will degrade localization accuracy. Therefore, Multi-stage AUV-aided Localization (MS-AUV) scheme is proposed in [52] aimed at improving MS-DNR scheme by replacing the DNR anchor node with an AUV. MS-AUV is introduced in Section 4.2.4. MS-AUV is proposed based on AUV-Aided localization technique, therefore, we first introduce the AUV-Aided localization technique in Section 4.2.3.

#### 4.2.4. AUV-Aided Localization Technique

AUV-Aided localization technique is proposed in [53], where an UWSN consists of many sensor nodes and one AUV. The sensor nodes are dropped into ocean and move with water currents. The AUV traverses the UWSN periodically following a predefined trajectory (a lattice-like and an Archimedean spiral trajectory). Moreover, all nodes can communicate (omni-directionally) with the AUV. The AUV can surface to obtain its coordinates by GPS, then dives to a predefined depth (provided by pressure sensors) and starts exchanging three types of messages with unknown nodes: wakeup, request and response. “Wakeup” messages are sent by the AUV as it enters the network to declare its presence to unknown nodes in its communication range. Unknown nodes that receive “wakeup” respond with a “request” message to commence range measurement. The “request/response” messages are exchanged between the AUV and unknown nodes to estimate their positions according to the round trip time. Then, localization is investigated using two methods, bounding-box [54] and triangulation.

**Figure 10.**
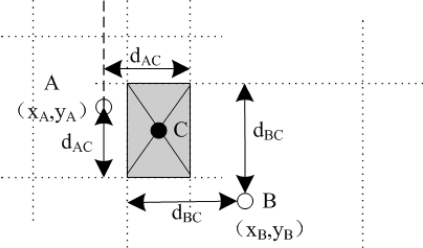
The Three-Dimensional Underwater Target Tracking.

Bounding box method draws a rectangular region with the intersection of the distance estimates (see Figure 10). The positions of unknown nodes are obtained by applying the distance measurements as constraints on the X and Y coordinates of unknown nodes. In Figure 10, *A* and *B* represent the positions of the AUV at two different time slots and *C* is the position of the unknown node. If the distance between the unknown node and the position of AUV *A* is *d_AC_*, then the *x* coordinates of node *C* are bounded by *d_AC_* to the left and to the right of the *x* coordinate of *A*, *x_A_ − d_AC_* and *x_A_* + *d_AC_*. The *y* coordinates of node *C* are bounded by *y_A_ − d_AC_* and *y_A_* + *d_AC_*. Similarly, the bounds for *C*’s coordinates with respect to B are obtained. The intersection of the diagonals gives the coordinates of the unknown node. The performance of bounding box is highly dependent on the positions of the AUV. An unknown node is better localized if the beacons are sent from opposite sides of the box. Compared with triangulation, bounding-box achieves a higher localization ratio but with a higher error.

AUV scheme does not assume any fixed infrastructure or time synchronization. Simulation results show that 100% localization can be achieved with only 3% localization error. However, localization time and accuracy are greatly influenced by the velocity and the location accuracy of the AUV. Since the velocity of the AUV is relatively slow, AUV-Aided localization technique has high localization delay (about up to 2 hours).

#### 4.2.5. Multi-Stage AUV-Aided Localization (MS-AUV) Scheme

To shorten localization time and improve localization accuracy of AUV-Aided localization technique, MS-AUV is proposed in [52]. MS-AUV combines the flexibility of the AUV-aided localization and the energy efficiency of “Silent Localization”, which has been studied in [26], where unknown nodes passively listen localization message. Simulation results show that the whole localization process can be completed in less than 10 minutes and can cover more than 95% of the whole network. However, similar to other multi-stage algorithms, e.g., MS-DNR, accumulated error is inevitable.

#### 4.2.6. Scalable Localization Scheme with Mobility Prediction (SLMP)

In [55], by utilizing the predictable mobility patterns of underwater objects, a Scalable Localization scheme with Mobility Prediction (SLMP) for UWSNs is proposed. Localization in SLMP is performed in a hierarchical way. The whole localization process is divided into two parts: anchor node localization and unknown node localization. During the localization process, every node predicts its future mobility pattern according to its past known location information. Moreover, every node can estimate its future location based on its predicted mobility pattern.

It is assumed that every sensor node needs to get its location periodically. During each localization period *T_p_*, anchor nodes can easily measure their locations since they can directly communicate with surface buoys. Based on the measured location *Loc_m_* in the last localization period and the predicted mobility pattern, each anchor node can calculate its estimated location *Loc_e_* in current localization period. The anchor node compares the estimated location *Loc_e_* with its measured location *Loc_m_*. If the Euclidean distance between them is larger than the stipulated threshold, the anchor node will judge that its current mobility pattern is not accurate and needs to be updated. Then, it runs its mobility prediction algorithm to get a new mobility pattern. After that, it will broadcast a new localization message which contains its current location and new mobility pattern to the network.

During unknown node localization process, all anchor nodes label themselves as reference nodes and set their confidence values to 1. When an unknown node receives localization information from anchor nodes, it runs its mobility prediction algorithm to estimate its own location and mobility pattern. If any unknown node has not received any localization message for a long period (larger than some predefined threshold), it will label itself as un-localized. With the advance of the localization process, more and more unknown nodes are localized and become reference nodes, as describe in [21,22].

Simulation results show that communication overhead and energy consumption of SLMP are relatively low. However, the performance of SLMP is easily influenced by the localization period *T_p_*. When *T_p_* is short, a relatively high communication overhead is needed. Furthermore, The performance of SLMP heavily depends on the structure of the mobility pattern.

### 4.3. Summary

As shown in Table 2, we compare the mobile localization algorithms from following aspects:

- Anchor type: In mobile localization algorithms, almost all anchor nodes are mobile except the stationary surface buoys used in MFALM and SLMP.
- Ranging method: Compared with one-way ToA, two-way ToA and TDoA alleviate the need for time synchronization. However, more message exchange is required and higher energy is consumed.
- Message communication: Unknown nodes stay “silent” in DNR, MS-DNR, AUV and MS-AUV to save energy.
- Time synchronization: In one-way ToA, accurate time synchronization is needed to estimate distances between anchor nodes and unknown nodes. Therefore, most algorithms require time synchronization except 3DUT, AUV and MS-AUV.
- Localization coverage: Most mobile localization algorithms have large localization coverage. However, localization coverage of APS and CLS are limited by the special network model.
- Localization time: In mobile UWSNs, when tracking unknown nodes or targets (APS, CLS and 3DUT), localization time should be as short as possible. Longer time used in EERS, MASL and DNR always introduce more energy consumption.
- Localization accuracy: In mobile UWSNs, localization accuracy of most algorithms is not high enough.
- Computational complexity: Computational complexity of mobile localization algorithms are relatively low.
- Energy consumption: Almost all mobile localization algorithms have high energy consumption for target tracking or hierarchical localization (MS-DNR, MS-AUV).

## 5. Hybrid Localization Algorithms

At present, some hybrid UWSNs has been employed where stationary and mobile sensor nodes coexist. For example, a hybrid architecture has been proposed in [56,57], where a mobile sink node traverses the network and collects data from underwater sensor nodes. In this section, hybrid localization algorithms are surveyed.

### 5.1. Centralized Localization Algorithms

In this section, two centralized localization schemes are researched: 1) 3D multi-power area localization scheme (3D-MALS) and 2) Silent Localization Using Magnetometers (SLUM).

#### 5.1.1. 3D Multi-Power Area Localization Scheme (3D-MALS)

In [58,59], ALS has been extended to 3D mobile UWSNs. The authors propose a range-free method based on mobile detachable elevator transceiver (DET) [60,61] and 3D multi-power area localization scheme (3D-MALS) to localize unknown nodes in deep underwater environment. In hybrid UWSNs, a small number of surface buoys are placed on the water surface. Each buoy is equipped with a DET, which is mainly composed of an elevator and an multi-power level acoustic transceiver. The elevator helps the DET rise or dive in vertical underwater, and the transceiver communicates with unknown nodes. The DET gets coordinate from its buoy when it moves to water surface, then moves down to broadcast position information at some pre-configured depths. At every broadcast position, the DET transmits beacon signals at varying power level as described in [12,13].

Unknown nodes listen to beacon signals periodically from the mobile DET. To save energy consumption of unknown nodes, all the measured position information are sent to the sink node to compute the estimation areas. However, the localization accuracy of 3D-MLS is low like that of ALS. Much energy is needed to transmit multi-power level signals. Moreover, localization time depends heavily on the velocity and message sending interval of the DET.

#### 5.1.2. Silent Localization Using Magnetometers (SLUM)

As discussed before, unknown nodes localization in UWSNs is traditionally addressed using acoustic range measurements involving known anchor or surface nodes. However, sound scatters significantly in underwater environments, especially in shallow water [62]. Therefore, magnetometers are often more useful than acoustics. For instance, Birsan has explored magnetometers and the magnetic dipole of a vessel for target tracking [63,64]. Dalberg *et al*. [65] fused electromagnetic and acoustic data to track surface vessels using underwater sensor nodes. In [66], Silent Localization Using Magnetometers (SLUM) is proposed. To the best of our knowledge, this is the first time magnetic dipole tracking is used to localize unknown nodes.

SLUM silently localize underwater unknown nodes equipped with triaxial magnetometers using a friendly vessel with known magnetic dipole. Unknown nodes are localized by listening to the messages of the dipole. The ferromagnetic field created by the dipole is measured by the magnetometers and is used to localize unknown nodes. Each unknown node is further equipped with a pressure sensor and an accelerometer used for depth estimation and sensor orientation estimation, respectively. The trajectory of the vessel and the positions of unknown nodes are estimated simultaneously using an Extended Kalman Filter (EKF) [66,67]. In SLUM, sensor nodes are assumed to be connected by wire. As a consequence, common problems in UWSNs such as time synchronization, limited bandwidth, and limited energy resources are neglected. However, the additional hardware requirement is the most drawback of SLM, hence, SLM is a costly localization method.

### 5.2. Distributed Localization Algorithms

In this section, four distributed localization schemes are researched: 1) An Range-free Scheme based on Mobile Beacons (RSMB), 2) Three-Dimensional Underwater Localization (3DUL), 3) Underwater localization based on directional beacons (UDB) and 4) Localization scheme with Directional Beacons (LDB).

#### 5.2.1. An Range-Free Scheme Based on Mobile Beacons (RSMB)

**Figure 11.**
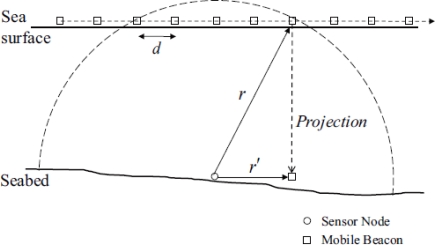
Range-Free Scheme Based on Mobile Beacons (RSMB).

An Range-free scheme based on Mobile Beacons (RSMB) is proposed in [68]. A mobile anchor node moves on the sea surface at a constant speed and broadcasts beacons at regular intervals, called beacon distance *d*. Here, the mobile anchor node follows the random way-point (RWP) model [69]; the mobile anchor node moves in a series of straight paths to random destinations. Unknown nodes are installed with pressure sensors to obtain depth information. As shown in Figure 11, the unknown node can receive five beacons from the mobile anchor node. Since the unknown node knows its own depth information, the received beacons can be projected on the plane where it locates. RSMB consists of two steps. The first step is to select three beacons among the received beacons. The second step is to estimate the location of unknown node.

In RSMB, each unknown node can estimate its own location independently. However, there are some problems of RSMB. First, localization time and accuracy mainly depend on the beacons’ sending interval. Second, high energy is consumed for the anchor node’s movement and the projection technology. Third, RSMB cannot localize unknown nodes in deep water. Furthermore, the authors do not research the path planning of the mobile anchor node, which severely affects localization accuracy.

#### 5.2.2. Three-Dimensional Underwater Localization (3DUL)

A three-Dimensional Underwater Localization (3DUL) is introduced in [70]. There are three buoys floating on the surface and many underwater sensor nodes are deployed at different depths. In addition, there are propelled autonomous robots freely floating with water currents. The underwater sensor nodes and the propelled robots are unknown nodes.

3DUL is a two phase protocol. During the first phase, unknown nodes estimates the distances to their neighbor buoys by using two-way message exchange technique and acquires their depth information. This phase of the algorithm is called Ranging. Once the distances to at least three buoys are estimated, the second phase of the algorithm, Projection and Dynamic Trilateration, is initiated. During this phase, the unknown node projects the buoys’ positions onto its horizontal level. Then, it localizes itself through trilateration and labels itself as a reference node. 3DUL does not require time synchronization. The biggest drawback of 3DUL is the very long localization time.

#### 5.2.3. Underwater Localization Based on Directional Beacons (UDB)

**Figure 12.**
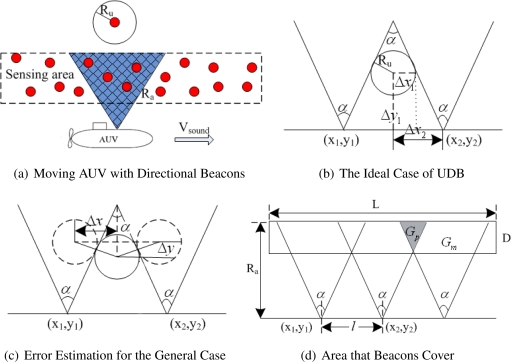
The Underwater Localization Approach Based on Directional Beacons (UDB).

Instead of traditional omnidirectional localization, the authors in [71] proposed a novel Underwater localization approach based on Directional Beacons (UDB) for hybrid UWSNs, where mobile AUV and stationary unknown nodes coexist. The directional beacons are transmitted by an AUV, which moves according to a predefined route navigated by compass, as shown in Figure 12(a). The sensing range of AUV is *R_a_*. A directional antenna is used on the mobile AUV and the angle of signal is controllable. For simplicity, two lines is used to imply the scope of the directional beacons in a 2D plane. The speed of the AUV is *V_sound_* and it is a stable value. *R_u_* is the sensing range of the unknown node. The distances in Figure 12(b) are expressed by equations listed as follow:

(1) $$\Delta x_{1}=\frac{R_{u}}{\text{cos}\left(\frac{\alpha }{2}\right)},\Delta x_{2}=\frac{x_{2}-x_{1}}{2},\Delta y_{1}=(\Delta x_{2}-\Delta x_{1})\times \text{cot}\left(\frac{\alpha }{2}\right)$$

(2) $$x=x_{2}-\Delta x_{2},\;y=y_{1}+\Delta y_{1}$$

However, the example in Figure 12(b) is the ideal case. The two edges of the beacon triangle are the tangent lines. Since beacons are not emitted continuously, some parts of the sensing area may not be covered. As illustrated in Figure 12(c), the biggest estimation error can be obtained as follows:

(3) $$\Delta x=2R_{u}\;\text{cos}\frac{\alpha }{2},\Delta y=2R_{u}\;\text{sin}\frac{\alpha }{2}$$

It is shown that if the sensing range *R_u_* is small enough, the ideal case of UDB can work in general case with an acceptable estimation error. When the AUV emits beacons within a fixed time interval, an optimization problem about the number of beacons is considered. As shown in Figure 12(d), the shadow part *G_p_* is the area that beacons can cover twice. Correspondingly, *G_m_* implies the area that beacons can cover once. The minimum number of beacons sent by the AUV can be optimized by minimizing *G_m_* and maximizing *G_p_*:

(4) $$G_{p}=\left\lceil \frac{L}{l}\right\rceil D^{2}\;\text{tan}\left(\frac{\alpha }{2}\right),G_{m}=\mathrm{LD}-\left\lceil \frac{L}{l}\right\rceil D^{2}\;\text{tan}\left(\frac{\alpha }{2}\right)$$

where,

(5) $$l=2(R_{a}-D)\;\text{tan}\left(\frac{\alpha }{2}\right)$$

The total numbers can be calculated by the following equation:

(6) $$N=\left\lceil \frac{L}{l}\right\rceil +1$$

To the best of our knowledge, this is the first time to introduce the idea of using directional beacons to localize unknown nodes in UWSNs. UDB is an range-free localization scheme that uses angle information to localize unknown nodes. However, localization coverage is limited. Hence, UDB is not suitable for large scale UWSNs. Moreover, if the AUV sends beacons with too long intervals, many unknown nodes may not be localized.

#### 5.2.4. Localization scheme with Directional Beacons (LDB)

**Figure 13.**
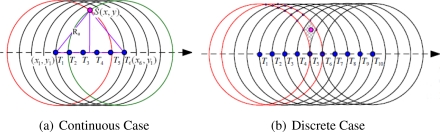
The Localization Scheme with Directional Beacons (LDB).

UDB is a two-dimensional localization. Luo *et al.* extended UDB into Sparse 3D Underwater environment and proposed a new Localization scheme with Directional Beacons (LDB) [72]. The depth of a node can be directly measured by a cheap pressure sensor, LDB is designed to determine its 2D position at the fixed depth. When AUV patrols at fixed depth of water following a straight line and sends beacons continuously, node *S* in its communication range can receive a series of beacons, as shown in Figure 13(a). Unknown node S hears the beacons when AUV sends them at *T*_1_*, T*_2_*, T*_3_*, T*_4_*, T*_5_ and *T*_6_, which are defined as mobile anchor points. *T*_1_ and *T*_6_ are the first-heard and the last-heard beacon points. The unknown node does not need to store all those six beacons. Only the first-heard and the last-heard beacon points are needed to localize node *S*. The position of *S* is thus calculated as:

(7) $$x=\frac{x_{1}+x_{6}}{2},y=y_{1}+\sqrt{R_{a}^{2}-{\left(\frac{x_{1}-x_{6}}{2}\right)}^{2}}$$

However, in practice, AUV sends beacons with time intervals. Nodes in the same small area will share the same first-heard beacon point and last-heard beacon point. As shown in Figure 13(b), four circles centered at point *T*_1_*, T*_2_*, T*_8_*, T*_9_ form a small area. If there are more than one unknown nodes residing in it, the unknown nodes share the same first-heard beacon point *T*_2_ and last-heard beacon point *T*_8_ with node S. Thus, using Equation (9) to localize unknown nodes incurs large localization error. Then, LDB uses the small area’s centroid to compute the positions of unknown nodes and estimate the upper bound of localization error.

### 5.3. Summary

As shown in Table 3, we compare the hybrid localization algorithms from following aspects:

- Anchor type: Most hybrid localization algorithms can localize unknown nodes in deep water by using mobile anchor nodes (DETs and AUV), using hierarchical localization method (3DUL) or other technology, such as magnetometer in LSUM. However, RSMB cannot localize deep water nodes by using one anchor node moving on the sea surface.
- Ranging method: Almost all the hybrid localization algorithms are rang-free except 3DUL.
- Message communication: In most hybrid localization algorithms, unknown nodes stay silent.
- Time synchronization: All the hybrid localization algorithms do not need time synchronization.
- Localization coverage: Only UDB and LDB have smaller localization coverage.
- Localization time: Due to mobile anchor node’s slow speed, localization time of 3DUL, DUB and LDB are relatively longer. The localization time of 3D-MALS and RSMB depends on the beacons’ sending interval.
- Localization accuracy: Due to sensor nodes’ unpredictable movement in hybrid UWSNs, all the algorithms cannot provide accurate location information.
- Computational complexity: Computational complexity of all the localization algorithms is low.
- Energy consumption: In general, active message communication costs more energy than silent one.

## 6. Summaries and Outlook

### 6.1. Summaries

In this paper, we give a comprehensive survey of the localization algorithms for UWSNs. From Table 4, we can see that each algorithm has its own characteristics and no one is absolutely the best. It is shown that:

- Although GPS does not work properly underwater and equipping sensor nodes with GPS is often expensive, most researchers still adopt anchor nodes to localize unknown nodes. Meanwhile, unknown node in sub-sea environment can be localized by the hierarchical approaches (e.g., LSHL, LSLS, MS-DNR, MS-AUV and 3DUL) or mobile anchor nodes (e.g., DETs, DNR anchor nodes and AUVs).
- In UWSNs, bandwidth constraint, sensor nodes’ mobility, and unpredicted variation in channel behavior make range methods based on received signal strength (RSS) and angle of arrival (AoA) inaccurate or unapplicable. Generally speaking, ToA-based or TDoA-based localization schemas are preferable even though they require time synchronization.
- In centralized localization algorithm, unknown nodes must stay “active” to transmit related localization information. In contrast, many distributed algorithms make unknown nodes passively listen to anchor nodes’ message, since nodes in silent communication spend much less energy and communication overhead.
- Most localization algorithms require time synchronization to estimate distances between unknown and anchor nodes. Due to nodes’ mobility, the mobile and hybrid localization algorithms often provide larger localization coverage than that of stationary localization algorithms, although they generally require longer localization time and give coarser location estimation.
- On the whole, the localization algorithms for UWSNs are computationally light. The energy consumptions of existing localization algorithms are generally high. Therefore, it is essential to design new energy efficient algorithms for UWSNs.

### 6.2. Outlook

In recent years, solving the localization problem in UWSNs has resulted in many innovative solutions and ideas. However, the research in this field is still at the start-up phase. The probable future research direction may be localization algorithms for mobile and hybrid UWSNs, since absolutely stationary network does not exist in real applications. We believe that in addition to the existing research issues of localization algorithms, the possible hot research topics are:

- Localization algorithms which are suitable for large scale UWSNs, especially for sub-sea environment, are still unexplored. At present, localization algorithms in UWSNs are mainly researched in the small scale network. Many practical applications require the use of large scale UWSNs. Therefore, it is essential to carry out the research of localization algorithms for large scale UWSNs.
- In UWSNs, developing an realistic model of sensor node’s mobility is an important issue. Many models, such as the Meandering Current Mobility (MCM) model, which has been studied in [48], have been proposed for mobility. However, these models are not suitable for sub-sea network. More efforts are needed to develop more realistic mobility models which can adjust anchor node’s movement path according to the corresponding underwater information, e.g., depth, current velocity, water salinity, *etc.*
- The issue of anchor node’s placement should not be neglected. Anchor node’s effective placement can greatly improve localization accuracy. For example, in the two-dimensional network, the localization error is least when three anchor nodes form an equilateral triangle.
- The present localization algorithms are lack of researches on anchor node’s path planning model in which the mobile anchor node traverses the entire network to provide larger localization coverage. In this case, the edge nodes and isolated nodes can be efficiently localized.
- Develop an efficient localization mechanism in which multiple anchor nodes dynamically collaborate with each other to localize unknown nodes. Especially needed is the research on how anchor nodes should collaborate to localize unknown nodes when the number of anchor node is not enough.
- The current localization algorithms are based on a safe and credible environment. However in real application, UWSNs are always deployed in complex and unsafe environment. Thus, secure localization and position verification algorithms are needed.
- To the best of our knowledge, there are few research on UWSNs’ localization problem under other conditions, such as duty-cycle environment, which is an interesting research issue. In duty-cycle environment, sensor nodes go to sleep according to some sleep scheduling mechanism. Therefore, current localization algorithms in which all sensor nodes wake up all the time cannot be adopted in the duty-cycle environment.
- Analysis of various influence factors is the premise of researching localization algorithms. It is important to design a realistic and systematic performance evaluation model and mechanism.

## Figures and Tables

**Table 1.** Comparison of Stationary Localization Algorithms.

Algorithms	Anchor type	Ranging method	Message communication	Time synchronization	Localization coverage	Localization time	Localization accuracy	Computational complexity	Energy consumption
ALS [12]	Stationary	Range-free	Active	No	Limited	Average	Low	Low	High
HLS [14]	Stationary	TDoA	Active	Yes	Medium	Average	High	Low	High
MLSL [15]	Sensor array	Range-free	Active	No	Large	Average	High	Low	High
PLM [17]	Stationary	Not specified	Active	Not specified	Medium	Average	High	Low	Low
ARTL [18]	Stationary	round-trip	Active	No	Large	Long	High	Low	High
NDLP [8, 9]	Anchor-free	Not specified	Active	Not specified	Large	Long	Low	High	High
LSHL [21,22]	Surface buoys, underwater anchors	One-way ToA	Active	Yes	Large	Long	Low	High	High
RLA [23]	Surface buoys	Not specified	Active	Not specified	Small	Long	Low	High	High
UPS [25, 26]	Stationary	TDoA	Silent	No	Small	Short	High	Low	Low
USP [29–31]	Stationary	Not specified	Silent	Not specified	Small	Short	Low	Low	High
LSLS [32]	Stationary	TDoA	Active	No	Large	Long	Low	Low	High
RBL [34]	Stationary	Two-way ToA	Active	No	Small	Average	High	Low	Low

**Table 2.** Comparison of Mobile Localization Algorithms.

Algorithms	Anchor type	Ranging method	Message communication	Time synchronization	Localization coverage	Localization time	Localization accuracy	Computational complexity	Energy consumption
APS [36]	GPS-positioned hydrophone	TDoA	Active	Not specified	Small	Short	Low	Low	High
EERS [39]	Anchor-free	One-way ToA	Active	Yes	Large	Long	Not specified	Low	High
MASL [40]	Anchor-free	One-way ToA	Active	Yes	Large	Long	Average	Low	High
CLS [42]	Anchor-free	One-way ToA	Active	Yes	Small	Short	Average	Low	Low
3DUT [43]	Float anchors	Two-way ToA	Active	No	Large	Short	Depend	Low	Low
MFALM [44]	Buoy nodes	TDoA	Silent	Not specified	Large	Long	High	Low	Low
DNR [45]	DNR	One-way ToA	Silent	Yes	Depend	Long	Depend	Low	Depend
MS-DNR [47]	Mobile	One-way ToA	Silent	Yes	Large	Short	Average	Low	High
AUV [53]	AUV	Two-way ToA	Silent	No	Depend	Depend	Depend	Low	High
MS-AUV [52]	AUV	Two-way ToA	Silent	No	Large	Short	High	Low	High
SLMP [55]	Surface buoys, underwater anchors	One-way ToA	Active	Yes	Large	Long	Low	High	Depend

**Table 3.** Comparison of Hybrid Localization Algorithms.

Algorithms	Anchor type	Ranging method	Message communication	Time synchronization	Localization coverage	Localization time	Localization accuracy	Computational complexity	Energy consumption
3D-MALS [58,59]	Surface buoys, DETs	Range-free	Silent	No	Large	Depend	Low	Low	High
SLUM [66]	Anchor-free	Range-free	Silent	No	Large	Short	Low	Low	Low
RSMB [68]	One mobile anchor	Range-free	Silent	No	Large	Depend	Depend	Low	High
3DUL [32]	Surface buoys	Two-way ToA	Active	No	Large	Long	Low	Low	High
UDB [71]	AUV	Range-free	Silent	No	Small	Long	Depend	Low	Low
LDB [72]	AUV	Range-free	Silent	No	Small	Long	Depend	Low	Low

**Table 4.** Comparison of UWSNs’ Localization Algorithms.

		Algorithms	Anchor type	Ranging method	Message communication	Time synchronization	Localization coverage	Localization time	Localization accuracy	Computational complexity	Energy consumption
		ALS [12]	Stationary	Range-free	Active	No	Limited	Average	Low	Low	High
		HLS [14]	Stationary	TDoA	Active	Yes	Medium	Average	High	Low	High
	Centralized	MLSL [15]	Sensor array	Range-free	Active	No	Large	Average	High	Low	High
		PLM [17]	Stationary	Not specified	Active	Not specified	Medium	Average	High	Low	Low
		ARTL [18]	Stationary	round-trip	Active	No	Large	Long	High	Low	High
Stationary		NDLP [8,9]	Anchor-free	Not specified	Active	Not specified	Large	Long	Low	High	High
		LSHL [21,22]	Surface buoys, underwater anchors	One-way ToA	Active	Yes	Large	Long	Low	High	High
		RLA [23]	Surface buoys	Not specified	Active	Not specified	Small	Long	Low	High	High
	Distributed	UPS [25,26]	Stationary	Two-way ToA	Silent	No	Small	Short	High	Low	Low
		USP [29–31]	Stationary	Not specified	Silent	Not specified	Small	Short	Low	Low	High
		LSLS [32]	Stationary	TDoA	Active	No	Large	Long	Low	Low	High
		RBBL [34]	Stationary	Two-way ToA	Active	No	Small	Average	High	Low	Low
		APS [36]	GPS-positioned hydrophone	TDoA	Active	Not specified	Small	Short	Low	Low	High
		EERS [39]	Anchor-free	One-way ToA	Active	Yes	Large	Long	Not specified	Low	High
	Centralized	MASL [40]	Anchor-free	One-way ToA	Active	Yes	Large	Long	Average	Low	High
Mobile		CLS [42]	Anchor-free	One-way ToA	Active	Yes	Small	Short	Average	Low	Low
		3DUT [43]	Float anchors	Two-way ToA	Active	No	Large	Short	Depend	Low	Low
		MFALM [44]	Buoy nodes	TDoA	Silent	Not specified	Large	Long	High	Low	Low
		DNR [45]	DNR	One-way ToA	Silent	Yes	Depend	Long	Depend	Low	Depend
		MS-DNR [47]	Mobile	One-way ToA	Silent	Yes	Large	Short	Average	Low	High
	Distributed	AUV [53]	AUV	Two-way ToA	Silent	No	Depend	Depend	Depend	Low	High
		MS-AUV [52]	AUV	Two-way ToA	Silent	No	Large	Short	High	Low	High
		SLMP [55]	Surface buoys, underwater anchors	One-way ToA	Active	Yes	Large	Long	Low	High	Depend
		3D-MALS [58,59]	Surface buoys,DETs	Range-free	Silent	No	Large	Depend	Low	Low	High
	Centralized	SLUM [66]	Anchor-free	Range-free	Silent	No	Large	Short	Low	Low	Low
Hybrid		RSMB [68]	One mobile anchor	Range-free	Silent	No	Large	Depend	Depend	Low	High
	Distributed	3DUL [32]	Surface buoys	Two-way ToA	Active	No	Large	Long	Low	Low	High
		UDB [71], LDB [72]	AUV	Range-free	Silent	No	Small	Long	Depend	Low	Low
